# Effects of puerarin on the intervertebral disc degeneration and biological characteristics of nucleus pulposus cells

**DOI:** 10.1080/13880209.2022.2147548

**Published:** 2022-12-16

**Authors:** Hengtao Tang, Song Zhang, Xinchang Lu, Tongyu Geng

**Affiliations:** a Department of Orthopaedics, The First Affiliated Hospital of Zhengzhou University, Zhengzhou, China; b Department of Orthopaedics, Shangqiu First People’s Hospital, Shangqiu, China

**Keywords:** Apoptosis, inflammation, extracellular matrix, toll-like receptor 4/nuclear factor-κB

## Abstract

**Context:**

Intervertebral disc degeneration (IDD) is the pathological basis of spinal degenerative diseases. Puerarin (PU) is an isoflavonoid with functions and medicinal properties.

**Objective:**

To explore the effect of PU on IDD and its potential mechanism of action.

**Materials and methods:**

Sprague-Dawley (SD) rats were divided into sham, IDD, low PU, and high PU groups. Rat nucleus pulposus cells (NPCs) were isolated and divided into control, IL-1β, 100 and 200 μmol/mL PU, TAK-242 (TLR4 inhibitor), or 200 μmol/mL PU + LPS (TLR4 activator) groups. The water content, inflammatory factors, proliferation activity, TLR4/NF-κB pathway activity, apoptosis rate, protein expression of apoptosis, and histology of the extracellular matrix (ECM) were analysed.

**Results:**

*In vivo*: Compared with the IDD group, disorganization of intervertebral disc tissue was significantly improved, water content (2.80 ± 0.24 mg, 3.91 ± 0.31 mg *vs.* 2.02 ± 0.21 mg) and expression levels of collagen II and aggrecan were significantly increased, and the levels of inflammatory factors and the expression levels of TLR4, MyD88, and p-p65 were significantly decreased in IDD rats treated with PU. *In vitro*: Compared with the IL-1β group, the proliferation activity of IL-1β-treated NPCs and the expression of collagen II and aggrecan were significantly increased, while the apoptosis rate, levels of inflammatory factors, and the expression levels of TLR4, MyD88, and p-p65 were significantly decreased in IL-1β-treated NPCs treated with PU. LPS reversed the biological function changes of IL-1β-treated NPCs induced by PU.

**Conclusions:**

PU can delay the progression of IDD by inhibiting activation of the TLR4/NF-κB pathway.

## Introduction

The intervertebral disc (IVD) is the largest nonvascular organ in the human body and is composed of nucleus pulposus (NP), annulus fibrosus (AF), and cartilaginous endplate (CEP) (Sun et al. 2020). NP cells (NPCs) play a key role in maintaining the biological characteristics and compressive strength of IVD by synthesizing and secreting collagen II and producing the extracellular matrix (ECM) of IVDs (Li et al. 2016). Intervertebral disc degeneration (IDD) is the pathological basis of a series of spinal degenerative diseases and is a common orthopaedic disease leading to decreased quality of life (Lv et al. 2014; Li et al. 2019). The aetiology of IDD is complex, and its exact pathogenesis is still unclear. At present, it is believed that the pathogenesis of IDD is mainly related to biomechanical factors, degeneration of NPCs, inflammation and degradation of ECM (Rui et al. 2020; Zhang et al. 2020a; Zhang et al. 2020b). Therefore, inhibition of inflammation, cell apoptosis and ECM degradation in NPCs may be therapeutic targets for delaying IDD.

**Figure 1.**
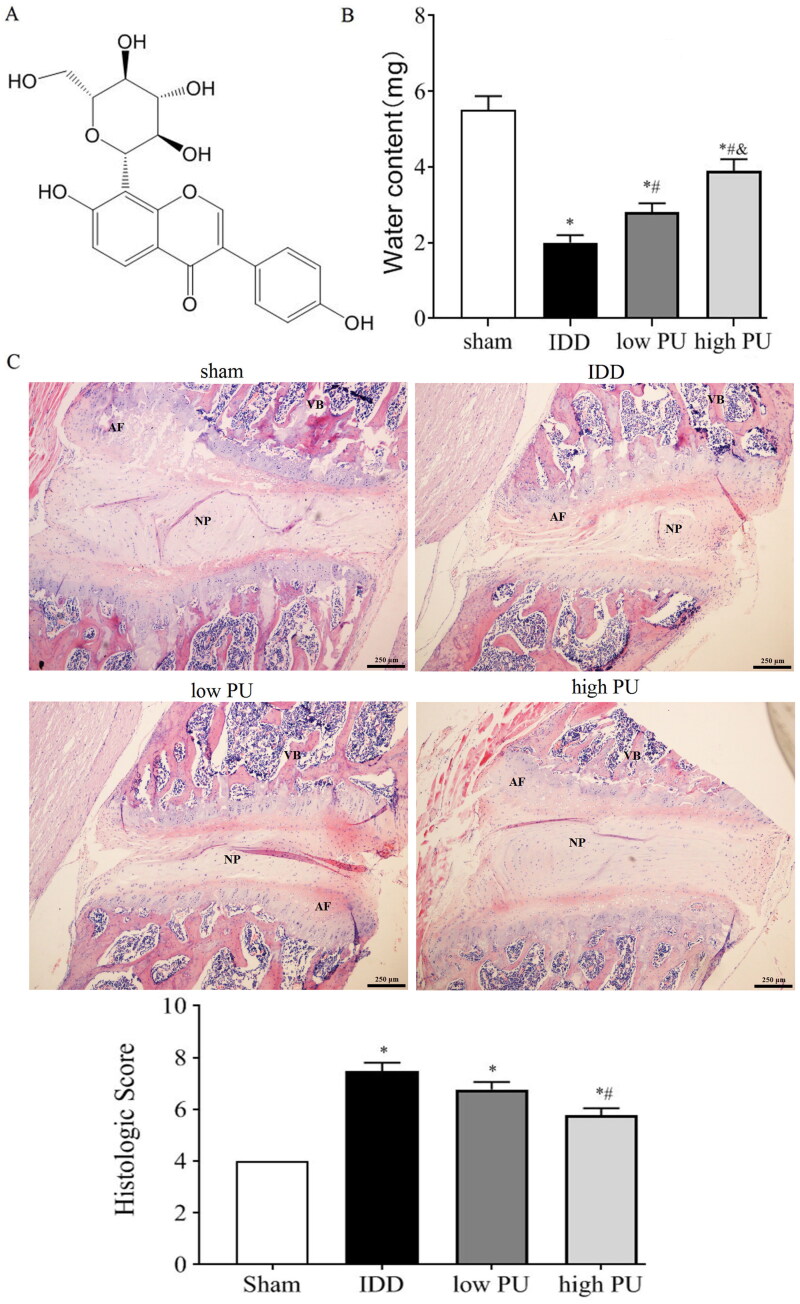
Effect of PU on water content and histopathology of IVDs in IDD rats. (A) Chemical structure of puerarin; (B) comparison of water content in IVD in each group; (C) pathological observation (HE staining, scale = 50 μm) Sham group. VB: vertebral body, NP: nucleus pulposus, AF: annulus fibrosus. Data are represented by mean ± SD, *n* = 9. *p < 0.05, *vs.* Sham group, *^#^p* < 0.05, *vs.* IDD group, *^&^p* < 0.05, *vs.* low PU group.

In recent years, compounds derived from natural products have shown promising results in treating degenerative disease with fewer side effects (Yang et al. 2019). Puerarin (PU, Figure 1(A)) is an isoflavonoid extracted from the Chinese medical herb Radix Puerariae Lobatae (also known as Gegen or Kudzu root) (Li et al. 2020). Recent pharmacology studies have shown that PU has many functions and medicinal properties, such as repairing endothelial cells, expanding the heart and cerebrovascular vessels, preventing osteoporosis and increasing antioxidative, antiapoptotic, and anti-inflammatory reactions (He et al. 2019; Jeon et al. 2020). In the clinic, it is used for the treatment of coronary cardiovascular diseases, lung diseases, and diabetes (Chen et al. 2021). Recently, some clinical and experimental data have demonstrated that intraarticular cavity injection of PU can reduce joint swelling, improve microcirculation, resist vasospasm, and improve blood hypercoagulability, thus playing a role in the treatment of osteoarthritis (Wang et al. 2018; Ma et al. 2020). Moreover, several studies have shown that PU reduces the expression of catabolic factors in IL-1β-stimulated chondrocytes, and can further suppress inflammation and ECM degradation by inhibiting the activation of NF-κB (Chen et al. 2021). Since NPCs are similar to chondrocytes in morphology and avascular supply (Yao and Flynn 2018; Mohanty et al. 2020), it is hypothesized that PU might attenuate the progression of IDD by protecting NPCs. The objective of this study was to investigate the effect of PU on IL-1β-treated NPCs and IDD rat models, and to further explore the potential therapeutic mechanism.

## Materials and methods

### Animals

A total of 40 male SD rats (200–220 g) aged 6–8 weeks, purchased from the Experimental Animal Centre of Zhengzhou University (quarantine licence No.: SCXK(Yu) 2017-0001). The animals were housed at 24 ± 1 °C under a 12 h light/dark cycle with free access to food and water. This experiment was approved by the Medical Research Ethics Committee of the First Affiliated Hospital of Zhengzhou University.

### Animal model

A total of 10 rats were treated with sham surgery: the skin tissue was only cut to expose the IVD and then sutured directly, regarded as the sham group. A total of 30 rats were randomly selected to generate models of IDD. The rats were anaesthetized by intraperitoneal injection of 5% chloral hydrate, placed in the supine position, and a right paracentral incision was made. The skin and subcutaneous tissue were incised successively. Subsequently, the upper and lower cartilage endplates of the lumbar IVD levels 4–6 (L4-6) were exposed. The above IVD segments were punctured with a 21 G micropuncture needle, which was inserted parallel to the cartilage endplate, and the depth of needling was equal to the whole layer of the AF. After the operation, the subcutaneous fascia and then skin were sutured successively. About 27 out of 30 rats successfully completed the model operation and were randomly divided into three groups with nine rats in each group: IDD group (intraperitoneal injection of 10 mL/kg d saline), low PU group (intraperitoneal injection of 100 mg/kg d PU) and high PU group (intraperitoneal injection of 200 mg/kg d PU). Intraperitoneal injection of saline or PU were treated at the same time every day for 8 weeks. After 8 weeks, the rats were sacrificed, and the L4-6 vertebrae were collected to conduct the following experiments. The L4-5 IVD section was used for water content determination.

### Measurement of water content in IVD

The water content in IVD was determined by vacuum freeze-drying. The cartilage tissues surrounding the whole L4-5 were removed and weighed, and the wet weight of the IVD was recorded. Then, the tissues were placed in a vacuum freeze-drying machine for 24 h to dry, and the dry weight of IVD was recorded. The water content in the L4-5 IVD sample of each group was calculated by subtracting wet weight from dry weight measurements.

### HE staining

The L5-6 sample of each group was fixed in paraformaldehyde, embedded in paraffin, and sectioned continuously (4 μm). The slices were dewaxed in xylene for 10 min, and then rehydrated with gradient alcohol. Then the samples were placed in haematoxylin dye solution for 15 min, and then dyed in 1% eosin solution for 10 min, washed, dehydrated, transparent in xylene, and sealed after air drying. The pathological sections were observed under a light microscope. Histologic score was calculated according to HE staining.

### Immunohistochemical staining

After dewaxing and gradient ethanol hydration, rabbit anti-rat TLR4 primary antibody (1:100) was added to the disc tissue sections, and the reaction time was 30 min at room temperature. After washing with PBS, HRP-labeled sheep-anti-rabbit secondary antibody (1:1000) was added and incubated at 37 °C for 30 min. After washing with PBS, DAB reagent was added for staining, drying, and sealing. The results were observed under a microscope. The positive staining result was brown or brownish-yellow granules in the cells. The degree of positive expression was evaluated by semi-quantitative analysis: the score of positive staining intensity from light to heavy was 1–3, and the score of no staining was 0. The score of the percentage of positive cells 0–5%, 6–25%, 26–50%, 51–75%, 76–100% was 0–4, respectively. The sum of the staining score and the percentage of positive cells was the final result.

### ELISA

The contents of TNF-α, IL-6, and IL-1β in IVD tissues and cells were detected by ELISA kits. The operation was performed in strict accordance with the ELISA kit (Beijing Solarbio Ltd. China) instructions.

### Western blot

Total protein extraction kit was used to extract the total protein of IVD tissues and cells. BCA method was used to detect the protein concentration. SDS-PAGE electrophoresis was used to transfer membrane and block. Primary antibodies (Abcam, UK) including anti-Bax (1:1000), anti-Bcl-2 (1:1000), anti-aggrecan (1:1000), anti-collagen II (1:5000), anti-TLR4 (1:1000), anti-MyD88 (1:500), anti-p-p65 (1:500), anti-Ki-67 (1:1000), and anti-GAPDH (1:1000) antibodies were added and incubated overnight at 4 °C. After washing the membrane, secondary antibody (1:5000) was added and incubated at room temperature for 1 h. ECL was added for developing. The software Image J was used for image analysis. GAPDH were used as internal parameters to calculate the relative protein expression according to the gray value of the band.

### Isolation and treatments of NPCs

The rat lumbar IVD tissue of the sham group was put into a petri dish containing PBS and cut to a size of 1 mm^3^. The tissue was digested with 0.1% collagenase II for 4 h at 37 °C, and then centrifuged at 1000 rpm for 5 min. The supernatant was discarded, and the cells were washed once with PBS, resuspended and counted in complete medium (DMEM/F12 medium containing 10% FBS and 1% penicillin–streptomycin). The cells were inoculated in a 25 cm culture bottle at a density of 1 × 10^6^ cells/mL, and placed in an incubator at 37 °C with 5% CO_2_ and saturated humidity. NPCs were passaged when cell confluence reached 80–90%, and the third generation of NPCs was used for subsequent experiments.

NPCs were divided into six groups: control group (conventional cultured without any treatment), IL-1β group (induced by 10 ng/mL IL-1β), 100 μmol/mL PU group (cultured with IL-1β and 100 μmol/mL PU), 200 μmol/mL PU (cultured with IL-1β and 200 μmol/mL PU), TAK-242 (a TLR4 inhibitor) group (cultured with IL-1β and 5 μM TAK-242), and 200 μmol/mL PU + lipopolysaccharide (LPS, TLR4 activator) (cultured with IL-1β, 200 μmol/mL PU and 0.5 μg/mL LPS). The cells were incubated with respective treatments for 24 h at 37 °C in 5% CO_2_. After 24 h of culture, further experiments detailed below were conducted.

### CCK-8 assay

Treated NPCs were inoculated into a 96-well plate (1 × 10^5^ cells/well) with five duplicate wells in each group. After 24 h of culture, 10 μL CCK-8 was added into each well and incubated at 37 °C for 2 h. The absorbance at 450 nm of each well was measured and cell viability was calculated with normal cells as a control.

### Flow cytometry

The cells in each group were digested by 0.25% trypsin, washed, and resuspended with pre-cooling PBS buffer to adjust the cell density to 1 × 10^5^ cells/mL. Annexin V-FITC (5 μL) was added and incubated in darkness for 15 min at room temperature. Then propidium iodide (PI) was added incubated for 5 min in darkness before the operation. The apoptosis rate of cells was detected by Cyto FLEX Flow cytometry (FCM).

The cells in each group were washed with PBS, and 70% precooled ethanol was added to fix the cells overnight at 4 °C. After washing with PBS again, 2 μL RNase A (0.25 mg/mL) and 500 μL PI dye solution (50 μg/mL) were added and dyed in dark at room temperature for 30 min. The cell cycle levels of each group were detected by FCM.

### Statistical analysis

All data were analysed by SPSS19.0. The one-way ANOVA analysis was used for the comparison among the groups, and the LSD-*t* test was used for the pairwise comparison among the groups. The value *p* < 0.05 means the difference was statistically significant.

## Results

### Effect of PU on the water content of IVDs in IDD rats

The water content in IVD was shown in Figure. 1(B). The water content in the IDD group was lower than that in the sham group (*p* < 0.05), while the water content in the low and high PU groups was significantly higher than that in the IDD group. The water content in the high PU group was significantly higher than that in the low PU group (*p* < 0.05). Those results indicated that PU could improve the decreased water content in IDD.

### Effect of PU on histopathology of IVD in IDD rats

HE staining showed that in IVD rat tissue, the AF and NP remained well organized, collagenous fibres were neatly arranged, and a large number of NPCs were noted in the sham group (Figure 1(C)). In the IDD group, the IVD tissue presented the characteristics of AF rupture, tissue texture disorder, and the reduction of NPCs to some degree, and some cells showed apoptotic-like changes. The histologic score of the IDD group was significantly higher than that of the sham group (Figure 1(C)). In the PU treated groups, NP structure was slightly more organized, fibrosis was relieved, and the number of NPCs was increased compared to the IDD group. The histologic score of the high dose PU treated was significantly lower than that of the IDD group (Figure 1(C)). These results suggested that PU could improve the degeneration of IVD.

### Effect of PU on inflammatory factors of IVD tissue in IDD rats

**Figure 2.**
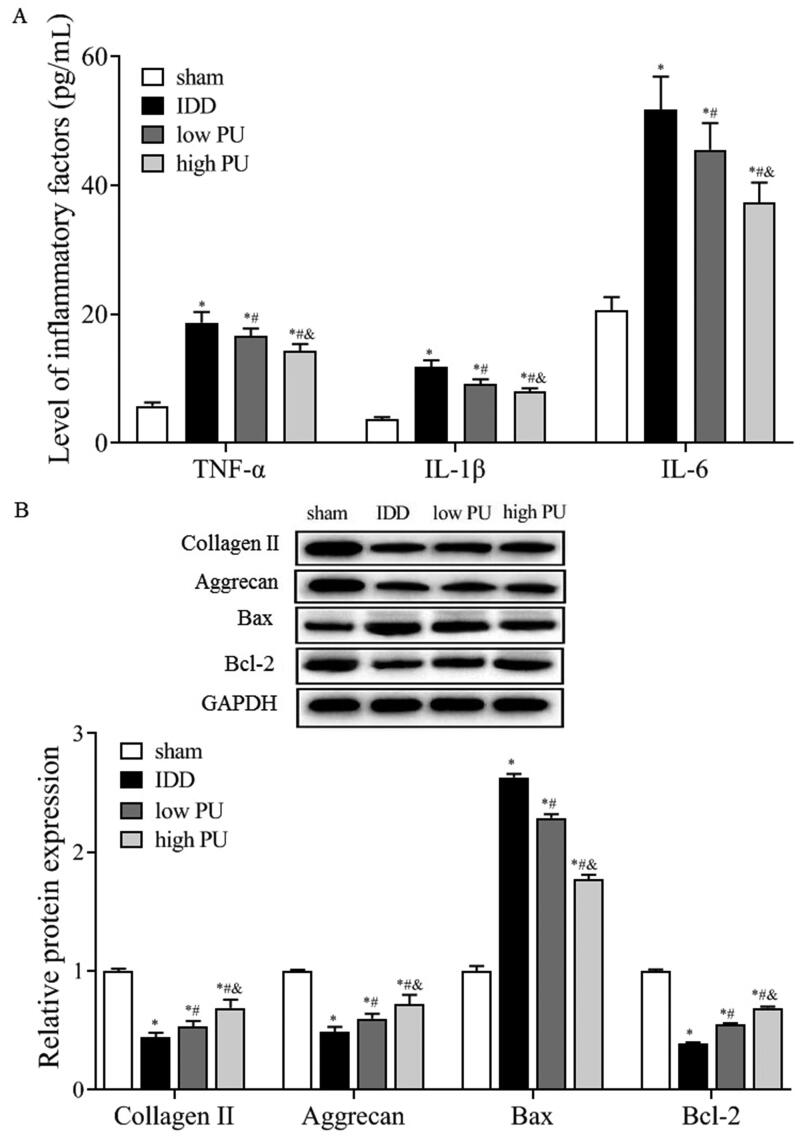
Effects of PU on inflammatory factors and ECM, apoptosis related proteins in IDD rats. (A) The contents of TNF-α, IL-6, and IL-1β in IVD tissues were analysed by ELISA; B: The protein expression levels of Collagen II, Aggrecan, Bax and Bcl-2 were analysed by western blot. Data are represented by mean ± SD, *n* = 9. *p < 0.05, *vs.* Sham group, *^#^p* < 0.05, *vs.* IDD group, *^&^p* < 0.05, *vs.* low PU group.

As shown in Figure 2(A), the concentrations of TNF-α, IL-1β, and IL-6 in the IDD group were significantly higher than those in the sham group (*p* < 0.05). Concentrations of inflammatory factors in the low and high PU groups were significantly lower than those measured in the IDD group (*p* < 0.05), and changes observed in the high PU group were statically significant (*p* < 0.05).

### Effects of PU on aggrecan and collagen II of IVD tissue in IDD rats

Western blot analysis showed that, compared with the sham group, the expression levels of collagen II and aggrecan were significantly decreased in the IDD group (*p* < 0.05). Compared with the IDD group (Figure 2(B)), the expression levels of collagen II and aggrecan were significantly increased in the low and high PU groups (*p* < 0.05), and changes in the high PU group were more obvious (*p* < 0.05).

### Effect of PU on apoptosis related proteins of IVD tissue in IDD rats

Western blot results showed that (Figure 2(B)), compared with the sham group, the expression level of Bax was significantly increased and the expression of Bcl-2 was significantly decreased in the IDD group (*p* < 0.05). Compared with the IDD group, Bax protein expression was significantly decreased and Bcl-2 protein expression was significantly increased in the low and high PU groups (*p* < 0.05), and changes in the high PU group were more obvious (*p* < 0.05).

### Effect of PU on TLR4/NF-κB signalling pathway in IDD rats

**Figure 3.**
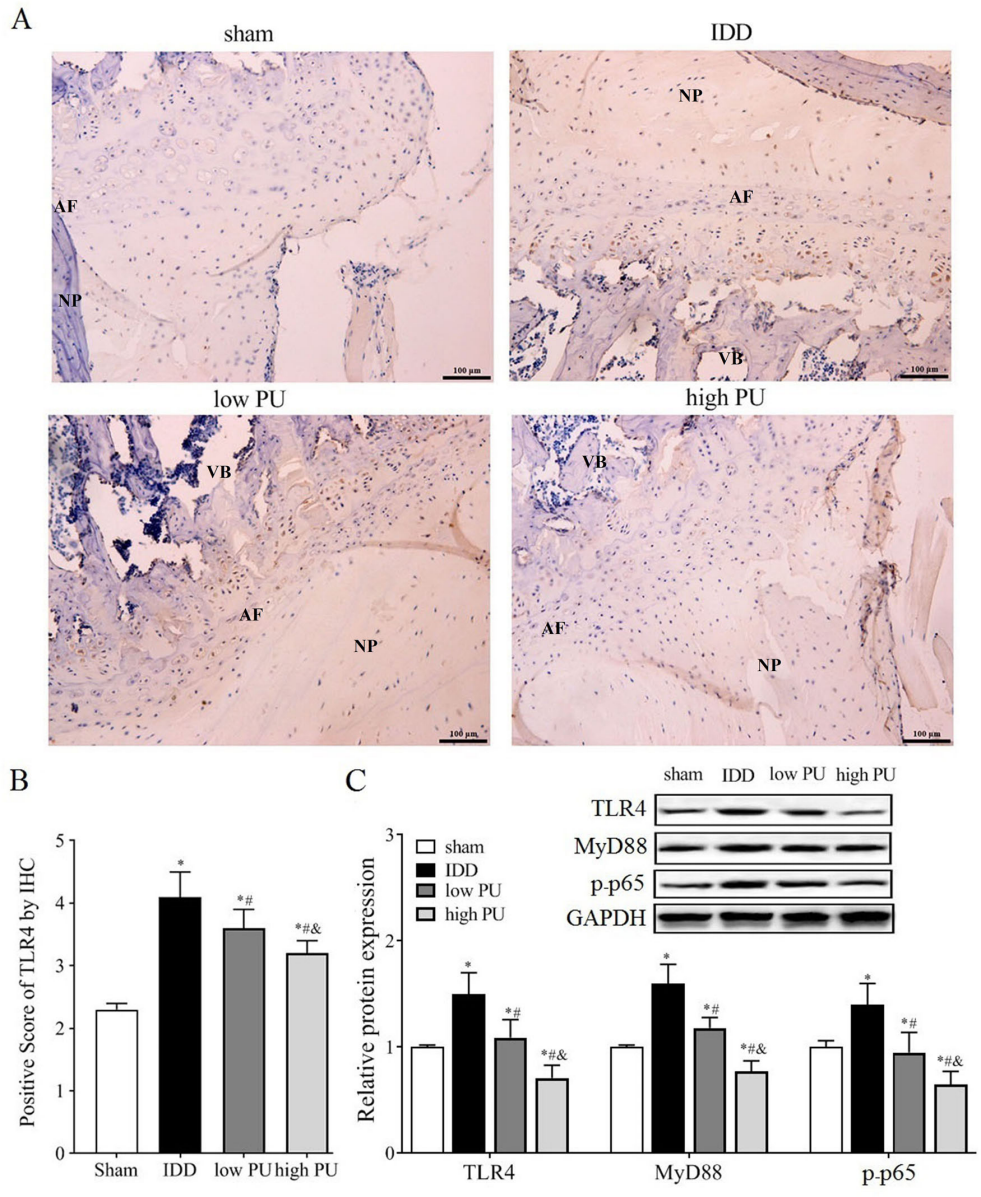
Effects of PU on TLR4/NF-κB signalling pathway in IDD rats. (A, B) IHC analysis of TLR4 expression in IVD tissue (scale = 100 μm). VB: vertebral body, NP: nucleus pulposus, AF: annulus fibrosus; C: The protein expression levels of TLR4, MyD88, and p-p65 were analysed by western blot. Data are represented by mean ± SD, *n* = 9. *p < 0.05, *vs.* Sham group, *^#^p* < 0.05, *vs.* IDD group, *^&^p* < 0.05, *vs.* low PU group.

The Immunohistochemical (IHC) assay (Figure 3(A, B)) showed that the main expression position of TLR4 protein is in the NP and AF. Compared with the sham group, the positive TLR4 score was increased in the IDD group (*p* < 0.05). Compared with the IDD group, the positive TLR4 score was decreased in the low PU and high PU groups, and the change in the high PU group was more obvious (*p* < 0.05).

Western blot analysis (Figure 3(C)) showed that compared with the sham group, the expression levels of TLR4, MyD88, and p-P65 were significantly increased in the IDD group (*p* < 0.05). Compared with the IDD group, the expression levels of TLR4, MyD88, and p-p65 were significantly decreased in the PU group (*p* < 0.05), and changes in the high PU group were more obvious (*p* < 0.05).

### Effects of PU on the viability of NPCs in vitro

**Figure 4.**
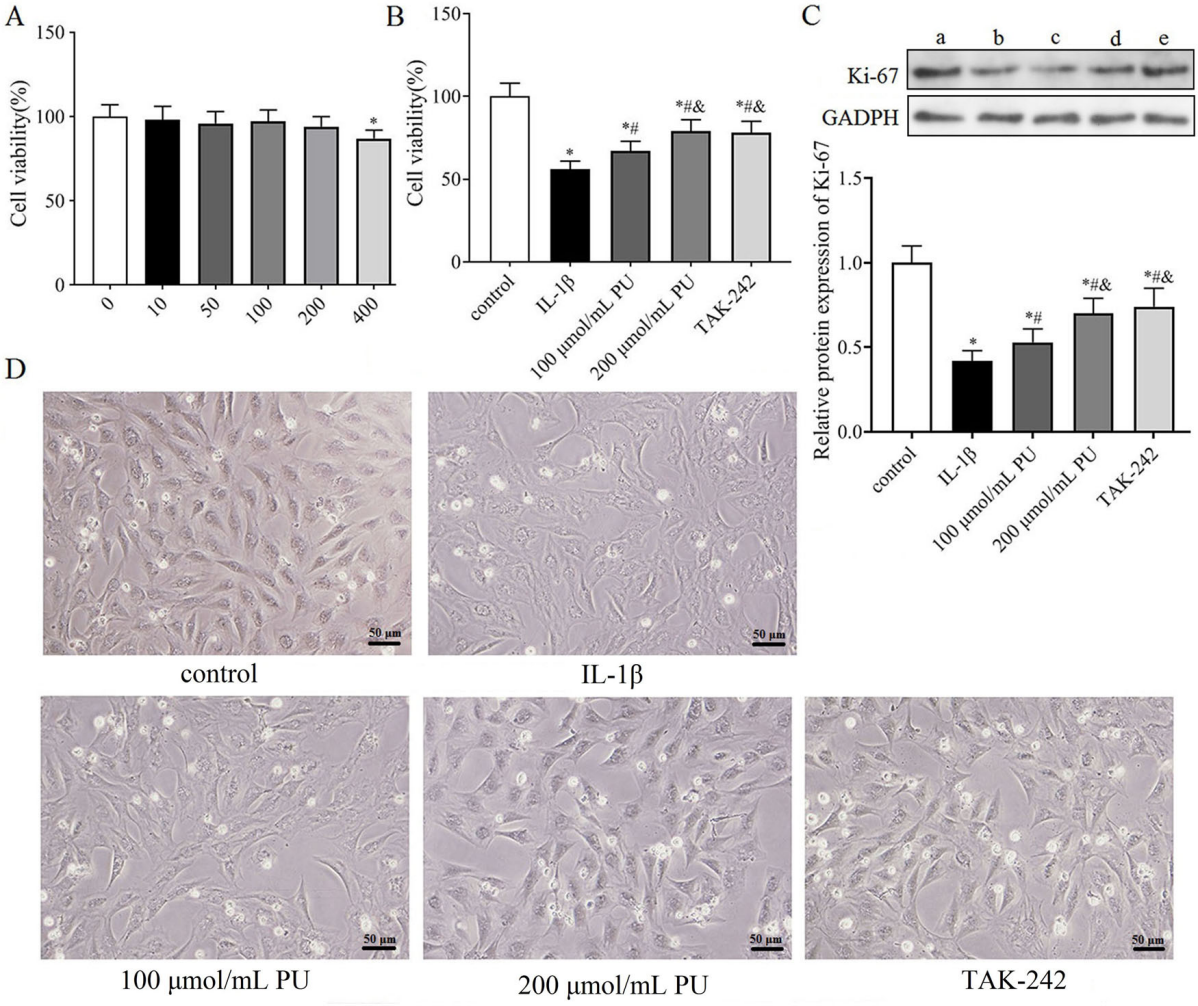
Effects of PU on the cell viability of NPCs. (A) The cytotoxic effect of PU on NPCs was determined at various concentrations for 24 h using a CCK8 assay; (B) effects of PU on cell viability of NPCs by CCK-8 assay; (C) The protein expression levels of Ki-67 were analysed by western blot (a: control group, b: IL-1β group, c: 100 μmol/mL PU group, d: 200 μmol/mL PU group, e: TAK-242 group); (D) The morphology of NPCs under optical microscope. Data are represented by mean ± SD, *n* = 3. *p<.05, *vs.* control group, *^#^p* < 0.05, *vs.* IL-1β group, *^&^p* < 0.05, *vs.* 100 μmol/mL PU group.

To evaluate the cytotoxic effect of PU on NPCs, the NPCs were treated with various concentrations (0,10, 50, 100, 200, 400 μmol/mL) of PU for 24 h. The CCK-8 assay showed that a PU concentration of 200 μmol/mL or less had no obvious cytotoxic effect on NPCs compared to the untreated cells; when the PU concentration reached 400 μmol/mL, the activity of NPCs was significantly inhibited (Figure 4(A)). Therefore, 100 and 200 μmol/mL of PU were used in the subsequent experiments.

To simulate the microenvironment of NPCs in IDD, IL-1β was used to stimulate NPCs to establish an IDD model *in vitro*. We then investigated whether PU treatment can alleviate the toxic effects of IL-1β on NPCs. The CCK-8 assay showed that IL-1β could significantly decreased the cell viability of NPCs, but PU could reverse this phenomenon (Figure 4(B)). Western blot analysis showed that the expression of Ki-67 in the IL-1β group was lower than that in the control group, while the expression level of Ki-67 in the 100 and 200 μmol/mL PU groups was higher than that in the IL-1β group, and changes in the 200 μmol/mL PU group were statistically significant (*p* < 0.05, Figure 4(C)). NPCs morphology were observed under the light microscope. Under the stimulation of IL-1β, the size of NPCs shrank, the cells outline was blurred, the boundary between nucleus and cytoplasm was unclear, and the overall number of cells were reduced, but under the effects of PU, the size and morphology of NPCs were improved, and the cell number was also increased (Figure 4(D)). These results suggested that PU showed significant protective effect on IL-1β-treated NPCs.

### Effects of PU on apoptosis of IL-1β-treated NPCs in vitro

**Figure 5.**
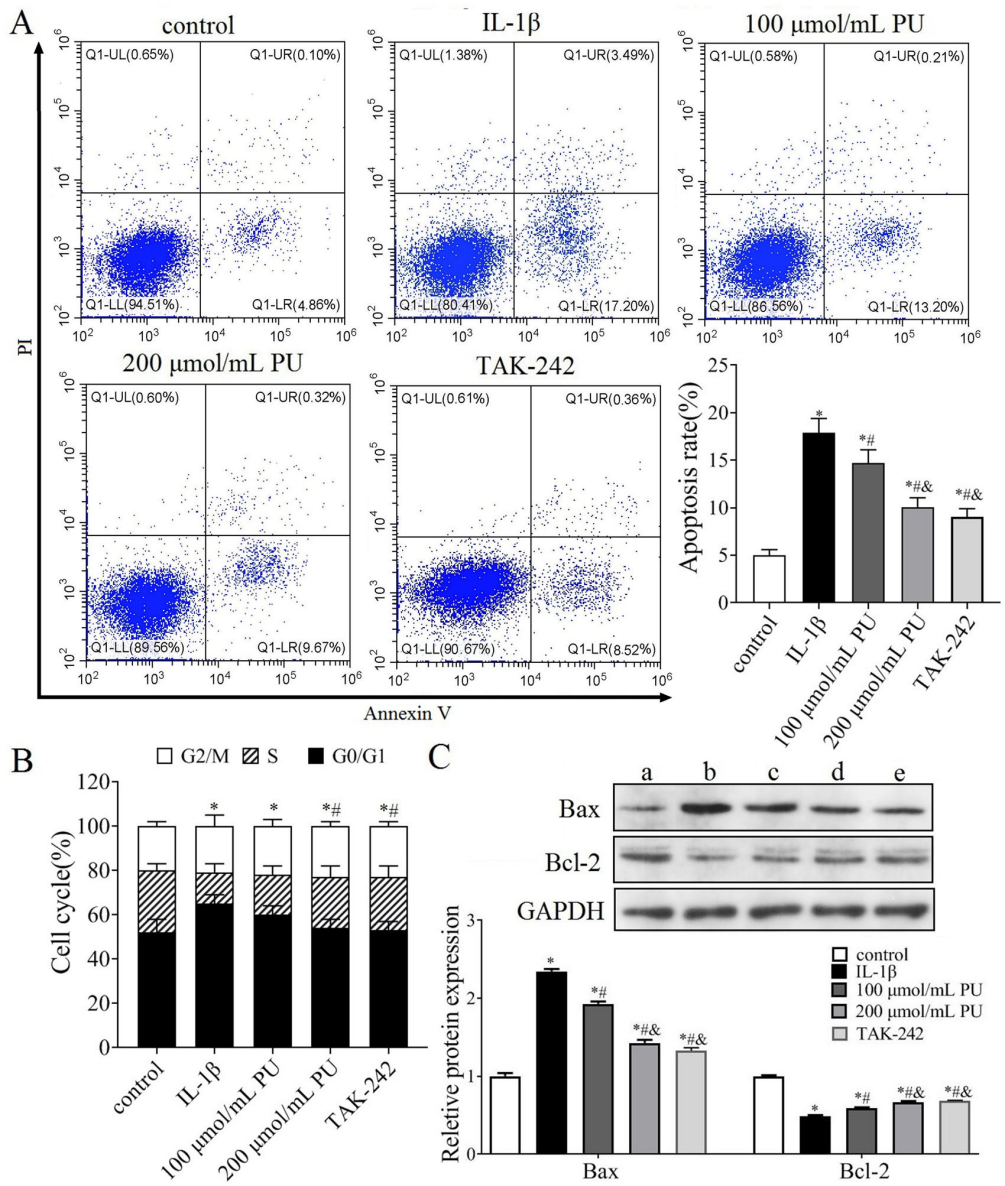
Effects of PU on apoptosis of IL-1β-treated NPCs. (A) The poptosis rate of NPCs detected by Annexi-V/PI double staining; (B) Effect of PU on the cell cycle of IL-1β-treated NPCs assessed by flow Cytometry; (C) The expression levels of Bax and Bcl-2 were analysed by western blot (a: control group, b: IL-1β group, c: 100 μmol/mL PU group, d: 200 μmol/mL PU group, e: TAK-242 group). Data are represented by mean ± SD, *n* = 3. *p < 0.05, *vs.* control group, *^#^p* < 0.05, *vs.* IL-1β group, *^&^p* < 0.05, *vs.* 100 μmol/mL PU group.

Abnormal apoptosis of NPCs also contributes to the progression of IDD. We therefore investigated the effect of PU on NPCs apoptosis. FCM analysis indicated that the apoptosis rate in the IL-1β group was higher than that in the control group (*p* < 0.05, Figure 5(A)). The rate of apoptosis measured in the 100 and 200 μmol/mL PU groups were lower than that of the IL-1β group, and changes in the 200 μmol/mL PU group were again significantly more obvious (*p* < 0.05, Figure 5(A)). The number of G1/G0 phase cells in the IL-1β group were higher than those in the control group (*p* < 0.05, Figure 5(B)), while the number of G1/G0 phase cells in the 200 μmol/mL PU groups were lower than those in the IL-1β group (*p* < 0.05, Figure 5(B)). Western blot analysis showed that compared with the control group, the expression of Bax was significantly increased, while the expression of Bcl-2 was decreased in the IL-1β group (*p* < 0.05, Figure 5(C)). Compared with the IL-1β group, the expression of Bax was significantly decreased, while the expression of Bcl-2 was increased in the 100 and 200 μmol/mL PU groups, and changes in the 200 μmol/mL PU group were more obvious (*p* < 0.05, Figure 5(C)).

### Effects of PU on the inflammatory factors in IL-1β-treated NPCs in vitro

**Figure 6.**
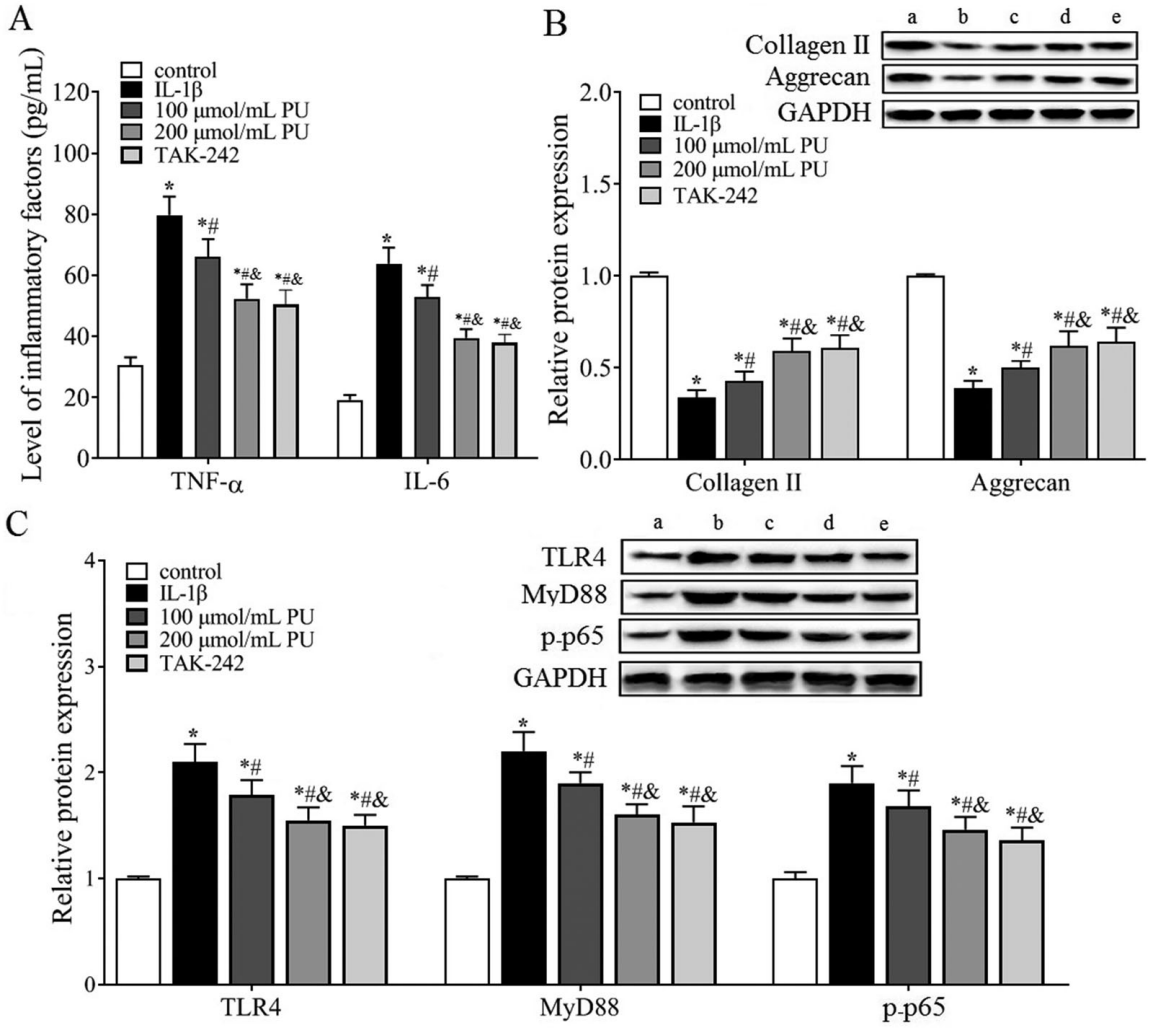
The effect of PU on inflammatory factors, ECM and TLR4/NF-κB signalling pathway in IL-1β-treated NPCs. (A) The contents of TNF-α and IL-6 were analysed by ELISA; (B,C) The expression levels of Collagen II, Aggrecan, TLR4, MyD88, and p-p65 were measured by Western blot (a: control group, b: IL-1β group, c: 100 μmol/mL PU group, d: 200 μmol/mL PU group, e: TAK-242 group). Data are represented by mean ± SD, *n* = 3. *p < 0.05, *vs.* control group, *^#^p* < 0.05, *vs.* IL-1β group, *^&^p* < 0.05, *vs.* 100 μmol/mL PU group.

ELISA (Figure 6(A)) showed that the concentrations of TNF-α and IL-6 in the IL-1β group were higher than those in the control group (*p* < 0.05), while those in the 100 and 200 μmol/mL PU groups were lower than those in the IL-1β group, and significantly more decreased in the 200 μmol/mL PU group (*p* < 0.05). These data suggested that PU treatment could suppress the enhancement of inflammatory cytokines IL-1β-treated NPCs.

### Effects of PU on collagen II and aggrecan in IL-1β-treated NPCs in vitro

Western blot analysis (Figure 6(B)) showed that, compared with the control group, the expression levels of collagen II and aggrecan were significantly decreased in the IL-1β group (*p* < 0.05). Compared with the IL-1β group, the expression levels of collagen II and aggrecan were significantly increased in the 100 and 200 μmol/mL PU groups, and these increases were more obvious in the 200 μmol/mL PU group (*p* < 0.05).

### Effects of PU on the TLR4/NF-κB signalling pathway in IL-1β-treated NPCs in vitro

**Figure 7.**
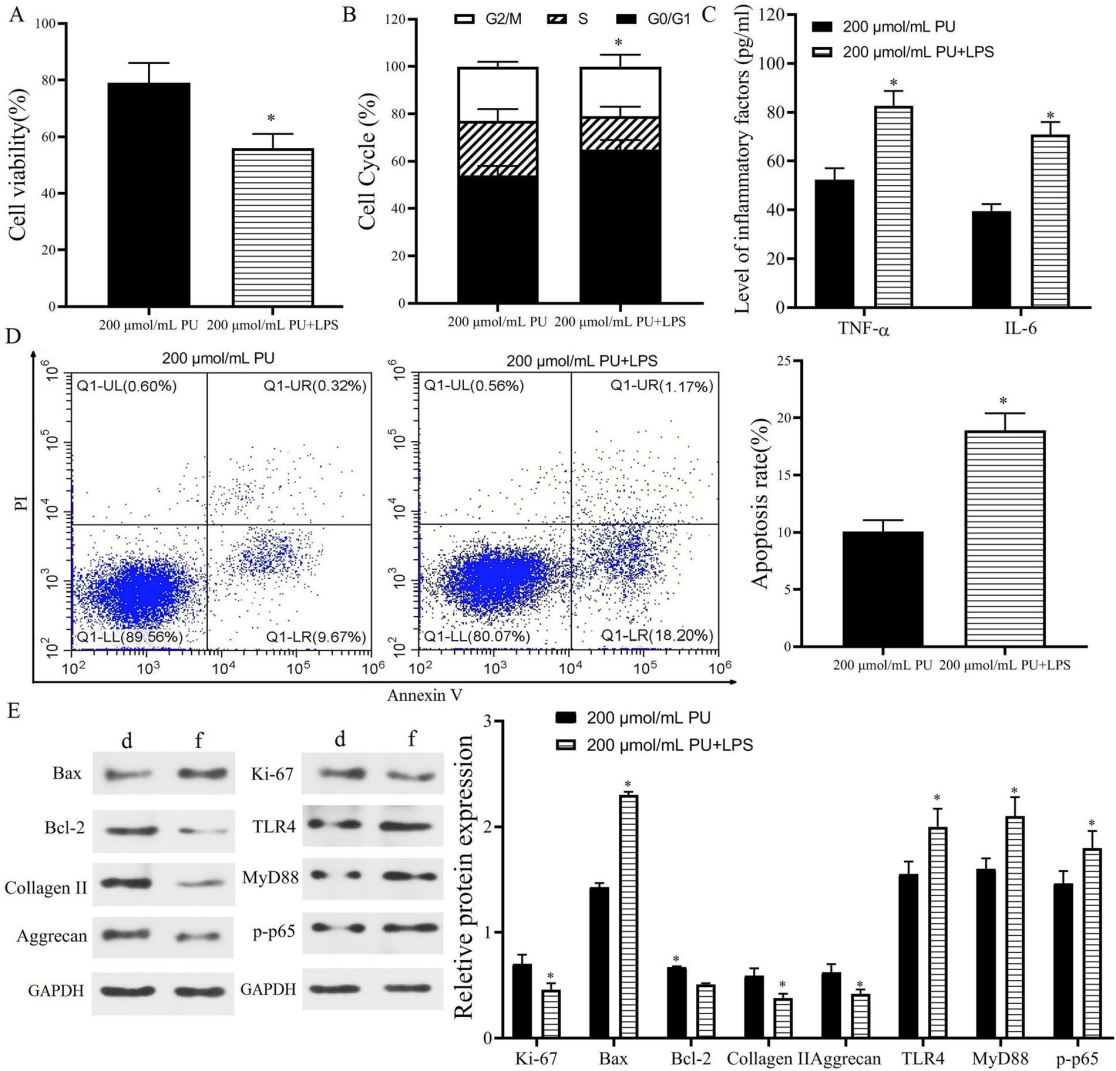
LPS reversed the effect of PU in IL-1β-treated NPCs. (A) The cell viability NPCs detected by CCK-8; (B) The cell cycle assessed by flow cytometry; (C) The contents of TNF-α and IL-6 were analysed by ELISA; (D) The poptosis rate of NPCs detected by Annexi-V/PI double staining; (E) The expression levels of Ki-67, Bax, Bcl-2, Collagen II, Aggrecan, TLR4, MyD88, and p-p65 (d: 200 μmol/mL PU group, f: 200 μmol/mL PU + LPS group). Data are represented by mean ± SD, *n* = 3. *p < 0.05, *vs.* 200 μmol/mL PU group.

The *in vivo* results showed that TLR4 and p-p65 proteins were highly expressed in IVD tissues of IDD rats, and PU intervention could downregulate their expression, suggesting that the TLR4/NF-κB pathway was involved in the pathogenesis of IDD and may be related to the mechanism of PU intervention in IDD. Therefore, we measured changes in the TLR4/NF-κB signalling pathway *in vitro* and used TAK-242 treatment as a positive control. Western blot analysis (Figure 6(C)) showed that compared with the control group, the expression levels of TLR4, MyD88 and p-p65 were significantly increased in the IL-1β group (*p* < 0.05). However, these parameters were significantly decreased after PU treatment, especially in the high PU group. There was no significant difference in the expression of TLR4, MyD88, and p-p65 between the TAK-242 group and the 200 μmol/mL PU group (*p* > 0.05). Similarly, the results of cell morphology observation under a light microscope, CCK-8 assay and FCM also showed that there were no differences in cell morphology, cell viability, or apoptosis rate between the two groups. These results suggested that PU restrained the activation of the TLR4/NF-κB signalling pathway in IL-1β-treated NPCs. To further clarify the mechanism of PU in IL-1β-treated NPCs, NPCs were treated with 200 μmol/mL PU and LPS. These results showed that the apoptosis rate, G1/G0 phase cells, the levels of TNF-α and IL-6, and expression levels of Bax, TLR4, MyD88, and p-p65 in the 200 μmol/mL PU + LPS group were higher than those in the 200 μmol/mL PU group (Figure 7). However, the cell viability and the expression of Ki-67, Bcl-2, aggrecan, and collagen II in the 200 μmol/mL PU + LPS group were lower than those in the 200 μmol/mL PU group (Figure 7). The above results indicated that LPS could reverse the biological function changes of IL-1β-treated NPCs induced by PU.

## Discussion

IDD is the main cause of a number of spinal diseases. ECM degradation, abnormal apoptosis of NPCs, and inflammation are the main pathological hallmarks of IDD. The ECM is mainly composed of collagen II and aggrecan proteins, and NPCs are the main source of both (Li et al. 2019). Therefore, the balance between growth and apoptosis of NPCs is the key to ECM balance and IDD. In addition, the increased expression of proinflammatory cytokines and inflammatory mediators, including IL-1, IL-6, IL-12, IL-17, TNF-α, and TNF-γ, is another feature of the IDD microenvironment (Penolazzi et al. 2019). Studies have shown that inflammatory cytokines such as IL-1β and TNFα increase the expression of MMP13, decrease the expression of collagen II and aggrecan, and induce NPCs apoptosis contributing to the degeneration of IVD (Hamamura et al. 2013; Lian et al. 2017). Therefore, the inhibition of inflammation, apoptosis, and ECM degradation of NPCs are considered the main therapeutic targets for delaying IDD.

PU has a variety of functional activities, such as anti-inflammatory, antioxidant, anti-osteoporosis, glucose-reducing, and anticancer activities. It is widely used in treatment for cardiovascular diseases such as angina pectoris, coronary heart disease, and myocardial infarction (Peng et al. 2019; Hou et al. 2021). Recent studies have shown that PU can inhibit inflammatory mediators, apoptosis, and ECM degradation in IL-1β-treated chondrocytes, alleviating the progression of osteoarthritis (Chen et al. 2021). Since NPCs and chondrocytes have similar phenotypes, it is speculated that PU also has a protective effect on chondrocytes (Chen et al. 2021). At the same time, PU has a certain protective effect on radicular pain from lumbar disc herniation and from transplantation of human NP mesenchymal stem cell (Zhong et al. 2018; Huang et al. 2020). Therefore, the effect of PU on IDD was currently studied *in vivo* and *in vitro*. Experiments *in vivo* showed that after treatment with PU, the IVD structure of IDD model rats were improved, the water content was increased, and the expression levels of ECM markers aggrecan and collagen II were increased, suggesting that PU could effectively alleviate the degeneration of IVD. Meanwhile, the results of *in vitro* experiments showed that PU could promote the cell viability of IL-1β-treated NPCs, promote the expression of aggrecan and collagen II, and reduce the apoptosis rate. These data support the idea that PU effectively improves the function of IL-1β-treated NPCs, promotes cell proliferation and ECM synthesis, and reduce the degradation of NPCs.

TLR4 is an important signalling molecule that mediates inflammatory response and initiates inflammatory immune response (Le et al. 2019). The MyD88-dependent pathway is the most important downstream signal transduction pathway of TLR4 (He et al. 2017). After extracellular binding, TLR4 and MyD88 can activate downstream effector factors to cause the activation and transport of NF-κB, further regulating the transcription of inflammatory mediators such as TNF-α, IL-6, and IL-1β, and playing an important role in cell apoptosis, tumour development, inflammation, and other physiological and pathological aspects (Hu et al. 2020; Liu et al. 2020; Zhang et al. 2020a). Current studies suggest that the TLR4/NF-κB signalling pathway plays an important role in the pathological development of IDD. Shen et al. (2019) found that the TLR4/NF-κB signalling pathway was generally activated in IDD patients, and the level of proinflammatory cytokines was increased. *In vitro* studies have also shown that LPS can activate the TLR4/NF-κB signalling pathway and induce an increase in proinflammatory factors. In this study, *in vivo* experimental results showed that protein expression of TLR4, MyD88, and NF-κB p-p65 in both IVD tissue of IDD rats and IL-1β-treated NPCs were significantly increased, which was consistent with previous findings (Wu et al. 2018; Zhang et al. 2018). Increasing evidence suggests that targeted inhibition of the TLR4/NF-κB signalling pathway has become one of the therapeutic strategies for IDD (Jacobsen et al. 2021). Su et al. (2019) found that isofraxidin can reduce the inflammation of IL-1β-treated NPCs by inhibiting NF-κB activation. Wang et al. (2019) found that *ligustilide* can inhibit the activation of NF-κB and reduce the expression of inflammatory factors, thus relieving IDD symptoms and reducing the incidence of IDD-related diseases. In addition, PU has also been found to have a protective effect on cisplatin nephrotoxicity by inhibiting the TLR4/NF-κB signalling pathway (Ma et al. 2017). Ni et al. (2020) found that PU could inhibit LPS-induced myocardial fibrosis by downregulating TLR4/NF-κB-related proteins, which suggested that PU has an inhibitory effect on the TLR4/NF-κB signalling pathway. However, it is not clear whether PU alleviates IDD by intervening in the TLR4/NF-κB pathway. In this study, the protein expression levels of TLR4, MyD88, and p-p65 in IDD rats after PU treatment were lower than those in the IDD rat model. Meanwhile, the *in vitro* results showed that PU inhibited the expression of TLR4, MyD88, and p-p65 in IL-1β-treated NPCs, and there was no difference between the high dose PU group and TLR4 inhibitor group. The TLR4 activator LPS reversed the biological function changes of IL-1β-treated NPCs induced by PU. Taken together, these results suggest that PU may delay the progression of IDD by inhibiting the activation of theTLR4/NF-κB signalling pathway.

## Conclusions

This study suggests that PU can decrease ECM degradation and inflammation, inhibit apoptosis, and delay the progression of IDD by inhibiting activation of the TLR4/NF-κB pathway. Therefore, PU may be a potential therapeutic option for the prevention and treatment of IDD.

## Data Availability

Data supporting the findings of this study are available from the corresponding author on request.
